# Pediatric thyroid cancer in Europe: time to act and rethink care

**DOI:** 10.1016/j.lanepe.2025.101551

**Published:** 2025-11-26

**Authors:** Sarah C. Clement, Hanneke M. van Santen

**Affiliations:** a Department of Pediatric Endocrinology, Wilhelmina Children's Hospital, University Medical Center Utrecht, Utrecht, the Netherlands; b Princess Máxima Center for Pediatric Oncology, Utrecht, the Netherlands

Pediatric papillary thyroid carcinoma (PTC) is a rare malignancy distinct from adult disease. In children, *RET*- and *NTRK*-fusions are more common, while *BRAF*-mutations predominate in adults.^1^ Pediatric tumors are typically larger, multifocal, and more metastatic, yet long-term survival is excellent, despite frequent recurrence or persistent disease.^2^

Historically, pediatric PTC management has mirrored adult protocols, typically involving total thyroidectomy and adjuvant radioactive iodine treatment (RAIT).^3^^,^^4^ While it can be effective in some cases, RAIT carries serious long-term risks, especially in children with longer life expectancy. Risk-adapted management could tailor surgery and RAIT use, minimizing overtreatment and exposure while preserving excellent disease-free survival.

Two recent randomized trials in *adults* have changed routine RAIT in low-risk PTC.^5^^,^^6^ The ESTIMABL2 trial randomized 776 low-risk patients (pT1a-b, N0/Nx, no extrathyroidal extension, no aggressive histology) to postoperative RAIT or no RAIT. Among 730 evaluable patients at three years, event-free survival was 95·6% without RAIT and 95·9% with RAIT, meeting the predefined non-inferiority margin.^5^ The recently published UK IoN 2025 trial (504 patients) confirmed and extended these findings to a broader low-risk cohort, including pT2 and selected pT3a tumors and N1a nodal disease. Five-year recurrence-free survival was 97·9% without RAIT vs 96·3% with RAIT, again demonstrating non-inferiority, with no increased recurrence risk even in higher-risk groups.^6^ By including pT3 and N1a cases, the IoN results are particularly relevant for pediatric cases, who often present with more advanced disease. Together these trials provide robust evidence, supporting recent adult PTC guideline revisions, demonstrating that RAIT can be safely omitted in carefully staged low-risk patients and emphasizing the urgent need to reconsider pediatric protocols.^7^

Observational North American studies provide insights on selective RAIT omission in *pediatric* PTC.^8^^,^^9^ Castellanos et al. followed 93 PTC patients, across risk groups who did not receive upfront RAIT when clinical factors suggested low benefit, including some higher-risk patients with no evidence of disease on postoperative dynamic risk stratification. Among the total cohort, 47 were node-negative, 35 without and 12 with upfront central neck dissection (CND). After a median 5·5-year follow-up, 91% remained disease-free, with slightly higher recurrence in those without CND.^8^ Bojarsky et al. studied 95 low-risk patients, 47% without RAIT. Median follow-up for the non-RAIT group was 3·6 years (range 0·9–9·3). One-year remission rates were similar between RAIT and non-RAIT patients (69% vs 70%), increasing with longer follow-up to 76% vs 82% (median 5·8 vs 3·6 years), with no persistent structural disease observed. CND was performed in 74.7% of patients overall, including 72% of RAIT patients and 78% of non-RAIT patients, many without pathologic nodal involvement (N0), suggesting prophylactic procedures.^9^

This may partly explain the apparent safety of RAIT omission. Outcomes could reflect surgery rather than RAIT omission, creating uncertainty for European practice, where prophylactic CND is generally avoided to reduce hypoparathyroidism and recurrent laryngeal nerve injury. Additionally, relatively short follow-up may bias results. These observational studies are hypothesis-generating and promising, but insufficient alone to change clinical guidelines.

This challenge is particularly urgent in Europe because the 2015 pediatric ATA and 2022 ETA guidelines differ substantially in treatment approach.^3^^,^^4^ In North America, many centers perform (prophylactic) CND and generally withhold RAIT in low-risk pediatric PTC, while in high-volume centers RAIT is also selectively deferred in some higher-risk patients based on postoperative dynamic risk stratification. Thus, RAIT decisions are guided not solely by pathology but also by reassessment.

In contrast, European practice largely avoids prophylactic CND yet continues to apply RAIT almost universally. Both strategies carry treatment-related risks, but the balance of harms and benefits remains unclear. Adult RCTs, cannot be directly extrapolated to children, and existing North American observational data are insufficient to guide adaptation of the current European pediatric guideline. Comparative analyses between European (ped-DTC registry)^10^ and North American (CATC) cohorts, alongside pediatric-specific RCTs, are urgently needed to develop evidence-based strategies that maximize cure while minimizing lifelong harm.

In the meantime, adherence to ETA-guidelines remains essential, with particular attention to low-risk patients. Clinicians should engage families in shared decision-making, carefully weighing the need for RAIT, considering selective or delayed use, and ensuring rigorous follow-up. This approach leverages current evidence to deliver effective care while minimizing avoidable treatment-related harm.

Looking ahead, insights from recent adult DTC-trials and North American pediatric studies call for immediate European action to optimize care. By leveraging risk-adapted strategies, registry-data, prospective studies, and international collaborations, clinicians can pinpoint which children truly need intensive treatment and which can safely avoid unnecessary RAIT or extensive surgery. Advanced imaging -particularly preoperative high-resolution ultrasound and functional cross-sectional modalities-combined with tumor biology, including somatic genotype and molecular profiling assessed pre- or postoperatively, can further refine risk-stratification. These efforts aim to safeguard survival and quality of life while minimizing harm. The time is now for clinicians and researchers to advance this field, delivering pediatric care that is thoughtful, precise, and grounded in the strongest evidence.

## Contributors

S.C.C. and H.M.vS. made equal contributions to this work. Both authors were involved in the conceptualization, design and structure of the Comment, literature search and review, drafting the manuscript, and revising it critically. Both authors approved the final version of the manuscript and are accountable for all aspects of the work.

## Declaration of interests

The authors declare that there are no conflicts of interest relevant to this work.
